# Antioxidant Compounds in the Treatment of Alzheimer's Disease: Natural, Hybrid, and Synthetic Products

**DOI:** 10.1155/2023/8056462

**Published:** 2023-02-21

**Authors:** Maryam Hatami, Mojtaba Mortazavi, Zahra Baseri, Batool Khani, Mehdi Rahimi, Sahar Babaei

**Affiliations:** Department of Biotechnology, Institute of Science and High Technology and Environmental Science, Graduate University of Advanced Technology, Kerman, Iran

## Abstract

Alzheimer's disease (AD) which is associated with cognitive dysfunction and memory lapse has become a health concern. Various targets and pathways have been involved in AD's progress, such as deficit of acetylcholine (ACh), oxidative stress, inflammation, *β*-amyloid (A*β*) deposits, and biometal dyshomeostasis. Multiple pieces of evidence indicate that stress oxidative participation in an early stage of AD and the generated ROS could enable neurodegenerative disease leading to neuronal cell death. Hence, antioxidant therapies are applied in treating AD as a beneficial strategy. This review refers to the development and use of antioxidant compounds based on natural products, hybrid designs, and synthetic compounds. The results of using these antioxidant compounds were discussed with the given examples, and future directions for the development of antioxidants were evaluated.

## 1. Introduction

Alzheimer's disease (AD), a neurodegenerative disorder, is associated with progressive neural loss, dementia, and other symptoms. Recently, due to population aging, the prevalence of AD for people over 65 had an adverse socioeconomic impact [1]. In spite of efforts, the exact etiologic of AD is not yet characterized [2]. There is evidence showing that some hidden players such as deficit of acetylcholine (ACh) [3], tau-protein aggregation [4], oxidative stress inflammation [5, 6], *β*-amyloid (A*β*) deposits [7], and dyshomeostasis of biometals [8] are involved in the progress of AD [9]. Among these, the amyloid *β* (A*β*) peptide fibrillization and senile plaques play a role in AD's pathological hallmarks [10]. Abnormal processing of amyloid precursor protein (APP) via *β*- and *γ*-secretases results in A*β* peptides which undergo conformational changes to generate organized *β*-sheet amyloid structures [11, 12]. Other studies have shown that increased biological markers related to oxidative stress, such as oxidized biological macromolecules (lipids, proteins, DNA, and RNA) [13] and 8-hydroxyguanosine (8-OHG) [14], as well as decreased antioxidant enzyme activity [15], were observed in the brain. In age-related disorders, oxidative stress produces peroxides and free radicals that induce cell death.

Reactive oxygen species (ROS) such as superoxide anion, hydroxyl radical (OH•), and hydrogen peroxide radicals (H_2_O_2_•) are produced in redox reactions [16–19]. Overproduction of ROS caused by higher consumption of O_2_ in the brain is responsible for the cognitive dysfunction observed in AD. The lack of balance between the prooxidant and the antioxidant elements is associated with protein oxidation and accumulation of A*β*. Increased ROS production would be able to impact the function of synapses. To reduce the rate of progression, antioxidant therapy has been suggested because of the prominent role of oxidative stress in AD [7, 20].

Various studies have indicated that antioxidants like vitamins E and C can be prolonged the progression of dementia [19]. Also, the nature of AD inspired the researcher to the development of multitargeted-directed ligands (MTDLs) which provide a more effective treatment than existing drugs [20, 21]. In a study conducted by Rossi et al., the MTDLs were selected based on hepatic, neuronal, and microglial cell toxicity [22]. In this study, the MTDLs were selected based on known pharmacophores in the natural products, hybrid designs, and synthetic compounds. Furthermore, we will review the role of antioxidant agents and their outcomes achieved in the treatment of AD.

### 1.1. Natural Product-Based Antioxidant Agents in AD

Natural products have been used to treat and reduce the progression of AD [21]. Extensive use of natural products due to less toxicity and fewer side effects makes them a very popular treatment [23]. The studies have confirmed the advantages of natural products such as vitamins C, E, luteolin, melatonin, curcumin [24], quercetin, resveratrol [25], huperzine A, and rosmarinic acid in the treatment of AD [26, 27].

### 1.2. Phenolic-Based Natural Compounds in AD

**Figure 1:**
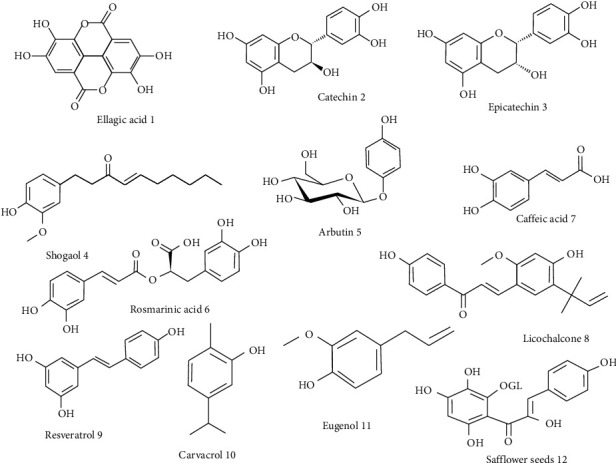
Natural compounds with phenolic structures.

Ferreres and coworkers have investigated the effects of ellagic acid and its derivatives against oxidative stress, AD, and depression. Since the phenolic compounds could be antioxidants and neuroprotective agents, the phenolic composition of aqueous and hydromethanolic extracts was determined by HPLC-DAD-ESI/MSn. The results illustrated that both extracts and ellagic acid had radical scavenging capacity compared to those of ascorbic acid. This indicated antidepressant activity while their anticholine esterase activities were weak [28] (Figure 1).

Cimini et al. evaluated the impact of the polyphenolic extract of cocoa against human AD. Cocoa polyphenols exerted their activity *via* triggering neuroprotection by activating the BDNF survival pathway, which led to the counteraction of neurite dystrophy, both on Aß plaque and Aß oligomers treated cells. Based on these results, cocoa powder could be used as a preventive agent for neurodegeneration [29]. For the development of polyphenolic compounds, Oligopin containing flavan-3-L units such as catechin (C) and epicatechin (EC) in pine bark extract have been identified (Figure 1). This polyphenol compound exerts its activity via blocking oligomer formation of A*β*1–40 and A*β*1–42, as well as tau in vitro [30].

In a similar study, Na et al. assessed the effect of 6-shogaol, an active constituent of ginger, with antioxidant and anti-inflammatory activities for the treatment of AD (Figure 1). It is suggested that A*β* (1–42) upregulated the cysteinyl leukotriene 1 receptor (CysLT1R) and cathepsin B. Results showed that 6-shogaol inhibited the CysLT1R and downregulated cathepsin B in both in vitro and in vivo models. Moreover, it decreases A*β* deposition in the brain of APPSw/PS1-dE9 Tg mice. For better results, one year later, this team evaluated the effect of 6-shogaol on sortilin-related receptor 1 (SORL1). Both in vitro and in vivo results indicated that the activation of SORL1 by 6-shogaol downregulates BACE, sAPPb, and A*β* as the preventive agent for the treatment of AD [31, 32]. The protective effects of the phenolic compound arbutin (from Pyrus biossieriana) with anti-inflammatory and antioxidant effects of streptozotocin (STZ)-induced neurotoxicity in rats have been compared. It is indicated that arbutin improves spatial memory and decreases oxidative and nitrosative stress. Overall, the neuroprotective effect of arbutin is associated with its antioxidants and free radical scavenging effects [33].

The inhibition of cholinesterase and antioxidant properties of various extracts of *Sideritis* albiflora and *Sideritis* leptoclada, which included rosmarinic acid and caffeic acid, were analyzed (Figure 1). The high antioxidant activity for the acetone extract of S. leptoclada in different assays such as *β*‐carotene‐linoleic acid (IC_50_: 17.23 ± 0.11 *μ*g/ml), DPPH• (IC_50_: 28.14 ± 0.05 *μ*g/ml), and ABTS (IC_50_: 15.18 ± 0.02 *μ*g/ml) assays, and the hexane extracts of sideritis species indicated moderate inhibitory activity on AChE and BChE [34]. Another multifunctional anti-Alzheimer agent is licochalcone B (LCB), as the main constituent of the root of *Glycyrrhiza* inflates (Figure 1). Cao et al. have evaluated the anti-AD activity of LCB through various tests. Based on the results, LCB could block amyloid-beta (A*β*_42_) self-aggregation (IC_50_ = 2.16 ± 0.24 *μ*M), disaggregate preformed A*β*42 fibrils, and decrease metal-induced A*β*42 aggregation via chelating metal ions. Also, upon exposure to LCB, the ROS generation was inhibited dose-dependently in SH-SY5Y cells, and the observed antioxidant activity of LCB was more potent than Cur [35]. The evaluation of antioxidant and neuronal cell protective effects of different fractions of *Erigeron* annuus leaf, including caffeic acid 7, showed that the highest antioxidant activity was related to butanol fraction because of the highest total phenolic contents (396.49 mg of GAE/g) and neuroprotective effects on neuronal cells [36].

Resveratrol (RSV) was isolated from *P*. *suffruticosa*, a member of a family of polyphenolic compounds called stilbenes (Figure 1). RSV reduces oxidative stress and has DPPH-free radical scavenging, as well as *β*-secretase inhibitory activity. The neuroprotective effect of this compound has been confirmed by regulating cholinergic, antioxidant, and anti-inflammatory pathways [37]. To develop functional foods for regulating glucose homeostasis and neuroprotection, Videira and coworkers conducted a study. They observed that a polyphenol-rich is extracted from polyvinylpolypyrrolidone (PVPP) winery residue with strong antioxidant activity blocks *α*-glucosidase (Ki = 166.9 *μ*g/mL) and aldose reductase (Ki = 127.5 *μ*g/mL). PVPP-white wine extract is capable of blocking rat brain AChE, decreasing ROS generation, and preserving the cell redox stat [38].

Kumar and coworkers have been investigating the neuroprotective mechanisms of clove oil in intracerebroventricular (icv)-colchicine-induced cognitive dysfunction in rats. The main constituents of clove oil include the phenolic compound carvacrol and eugenol (Figure 1). After the administration of clove oil, an impaired cognitive performance in the Morris water maze (MWM) was observed that results in oxidative stress increased AChE level, and neuroinflammation and mitochondrial dysfunction. The main neuroprotective effect of clove oil may be partly because of its mitochondrial restoring and antioxidant properties along with a microglial inhibitory mechanism [39].

Cholinergic dysfunction and oxidative stress are involved in AD. Safflower seeds with phenolic structure have a reputation as an antioxidant agent that could improve cholinergic compounds like serotonin (Figure 1). Cho and coworkers investigated the effects of safflower seed extract on scopolamine-induced memory impairment in a mouse model. The results showed that safflower seed extract could improve memory function via inhibition of the AChE activity. In comparison, the safflower seed decreases ROS production and increases antioxidant enzyme levels, indicating the protective role of the safflower seed extract against oxidative stress [40].

### 1.3. Salvianolic Acid and Flavonoid Natural Compounds in AD

**Figure 2:**
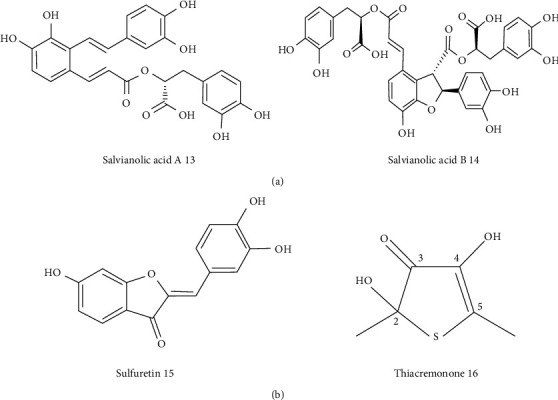
(a) Salvianolic acid in the treatment of AD. (b) Flavonoid compounds in AD.

There is evidence that metal ions generate oxidative damage to neuronal cells. Cao et al. studied the effects of salvianolic acid A (Sal A) on the treatment of AD (Figure 2(a)). Obtained results showed that Sal A blocks amyloid-beta (A*β*), self-aggregation and disaggregates preformed A*β* fibrils, decreases metal-induced A*β* aggregation, and prevents the formation of ROS in SH-SY5Y cells [41]. Many Chinese herbs with antioxidant properties have been regarded as neuroprotective agents. Gazova and coworkers selected salvianolic acid B (Sal B), which is extracted from Salvia miltiorrhiza and anemarrhenae asphodeloides to evaluate their dissociation potential towards A*β*42 peptide fibrils and neuroprotective effect (Figure 2(b)). Most compounds could be able to dissociate A*β*42 fibrils. One of the sarsasapogenin derivatives showed a decreased level of nitride oxide production [42].

The neuroprotective effects of sulfuretin 15 (Figure 2(b)), a member of flavonoid glycosides with antioxidant properties which are isolated from the stem bark of Albizzia julibrissin and heartwood of Rhus verniciflua, have been investigated by Kwon et al. They found that sulfuretin decreases the release of lactate dehydrogenase and accumulation of ROS associated with A*β*25–35-induced neurotoxicity in neuronal cells. It upregulates the expression of heme oxygenase-1 (HO-1) and protects neuronal cells from A*β*25–35-induced neurotoxicity via activation of Nrf/HO-1 and PI3K/Akt signaling pathways [43]. Various studies show that the accumulation of amyloid-beta (A*β*) peptide in AD is related to oxidative stress and inflammatory responses. Hence, Yun et al. investigated the neuroprotective effects of thiacremonone isolated from garlic, which has antioxidant and anti-inflammatory properties (Figure 2(b)). Thiacremonone could be able to improve memory by the stimulating antioxidant system when it upregulated the expression of peroxiredoxin 6 (PRDX6) for reducing oxidative stress of macromolecules such as protein and lipids. Exposure by thiacremonone, H_2_O_2_, and A*β*1–42 in embryonic neuronal cells inhibited the activation of NF-*κ*B and ERK pathways [44].

### 1.4. Other Structures in Natural Compounds in AD

Hritcu and coworkers have assessed possible anxiolytic, antidepressant, and antioxidant properties of *F*. *angulata* essential oil, which is used for treating digestive pains, haemorrhoids, snake bites, and ulcers. In the scopolamine-induced rat model of Alzheimer's disease, the results showed anxiolytic, antioxidant, and antidepressant-like effects. It is proposed that *F*. *angulate* essential oil inhalation relieves scopolamine-induced anxiety and depression by the decrease of oxidative stress in the rat amygdala [45].

**Figure 3:**
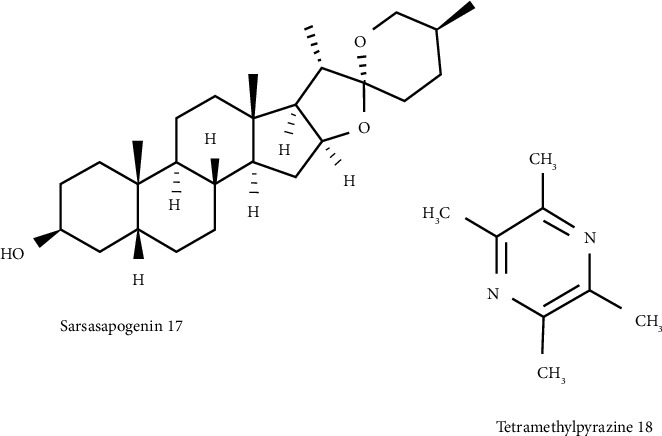
Other structures in natural compounds.

Today, using a multitarget-directed ligand (MTDL) strategy, the design of a new indole drug, synthesis of novel chromone+ donepezil hybrids, and development of carbazole scaffold based as potential anti-Alzheimer agents were conducted [46–49]. To develop a therapeutic strategy against AD by using an MTDL approach, the antiamyloidogenic, antioxidant, and neuroprotective properties were found in the *Asparagus racemosus* aqueous extract and its secondary metabolite sarsasapogenin (SRS) 17 (Figure 3). SRS dose-dependently inhibits AChE, BuChE, BACE1, and MAO-B as well as blocks A*β*42 fibrillization up to 68%. In addition, the SRS indicated a neuroprotective effect on PC12 cells against A*β*42 and H_2_O_2_-mediated cytotoxicity. Thus, SRS could be served as an MTDL compound against AD [50]. To relieve the inflammatory progression of AD, Lee and coworkers found tetramethylpyrazine (TMP) 18 (Figure 3).

TMP is able to block the A*β*25–35 fibrillization and production of nitric oxide. This compound was an example of controlling A*β*-related neuropathology [51]. For stress-related disease prevention, herbs such as *Ipomoea* aquatica Forsk (HAEIA) were used to diminish oxidative stress in the brain. The results of Sivaraman and coworkers indicated that HAEIA significantly upregulates the expression of the enzymes, including superoxide dismutase (SOD), catalase (CAT), glutathione peroxidase (GPX), and glutathione reductase (GPR). HAEIA prevents the progression of DNA damage and as a potential antistress compound could be applied in AD [52].

### 1.5. Hybrid-Based Antioxidant Agents in AD

Since the nature of AD includes a complex cascade of molecular events in the brain, multifunctional drugs could be beneficial. Conjugation of the AChE inhibition and antioxidant properties in a molecular hybrid is known as an effective strategy.

## 2. Phenolic Acids-Based Hybrids

Šebestík et al. hybrid the naturally occurring phenolic acid compounds (derivatives of caffeic acid, rosmarinic acid, and trolox), with choline to account for the recognition by AChE. Most of the hybrids indicated AChE inhibitory activity and good antioxidant properties [53]. Curcumin which is extracted from plant rhizomes “Curcuma longa Linn” has anti-inflammatory and antioxidant activities and scavenges free radicals while protecting the brain from lipid peroxidation.

### 2.1. Curcumin-Based Hybrids

For the design of hybrid drugs, Elmegeed et al. combined the promising heterocyclic nucleus with the essential pharmacophoric features of the curcumin moiety. This hybrid compound decreases AchE activity, urinary 8-OHG level, serum caspase-3 level, and brain P53 level relative to the control group [54].

### 2.2. Donepezil-Based Hybrid

**Figure 4:**
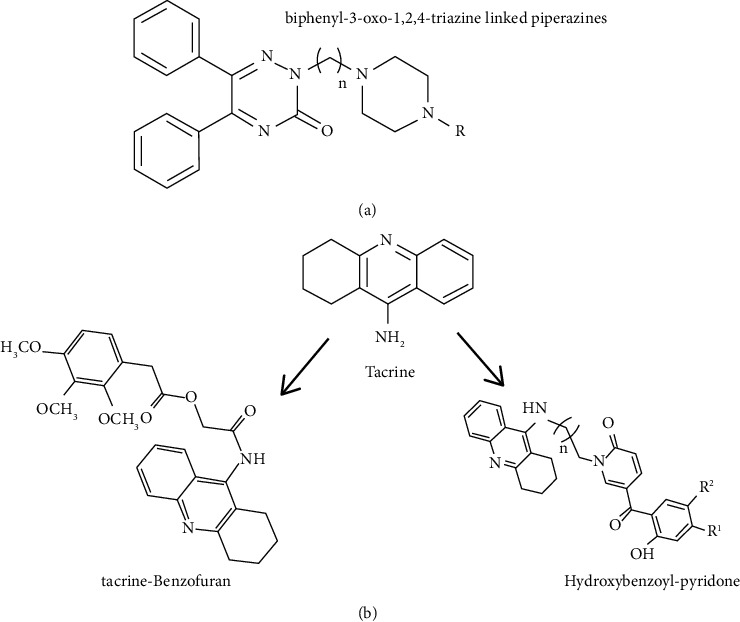
(a) Structure of biphenyl-3-oxo-1,2,4-triazine linked piperazine derivatives 19. (b) Structures of tacrine-based hybrids 20 and 21.

Using a hybrid strategy for the design of multifunctional anti-AD agents, Tripathi et al. introduced biphenyl-3-oxo-1,2,4-triazine-linked piperazine derivatives (see Figure 4(a)). They connected biaryl-1,2,4-triazines with intrinsic antioxidant property with the piperazine moiety as a bioisostere of the piperidine ring in donepezil, an FDA-approved drug against dementia, via the different lengths of alkyl linkers. The most active compounds in this series were able to inhibit AChE (IC_50_: 0.2 ± 0.01 *μ*M) more effectively than standard donepezil (IC_50_: 0.1 ± 0.002 *μ*M). Also based on ex vivo studies, AChE inhibition and reversal of the scopolamine-induced oxidative stress were observed [55].

The novel tacrine-(hydroxybenzoyl-pyridone) (TAC-HBP) hybrids have been designed and synthesized by Santos and coworkers. All the hybrid compounds inhibit AChE in the submicromolar range (IC_50_ = 0.57–0.78 *μ*M) compared to the parent tacrine, the well-known AChE inhibitor, and showed good DPPH radical scavenging capacity (EC_50_ = 204–249 *μ*M) conferred by the hydroxybenzoyl-pyridone (HBP) moiety. The most active compound chelates of various biometals pFe = 13.9, pCu = 6.0, and pZn = 6.0, at pH 6.0, CL/CM = 10, and CM = 10−6 M [56]. Also, this group conjugated tacrine with benzofuran (BF) to develop a single agent which inhibits AChE and amyloid-beta peptide aggregation as well as chelate metals (Fe, Cu) and associated extra antioxidant activity. TAC-BF hybrids showed the submicromolar range of AChE activity while could able to inhibit self- and Cu-mediated A*β* aggregation (Figure 4(b)) [57].

Using tacrine, trolox, and *β*-carboline derivatives with versatile biological activities as promising leads for developing AD, the tacrine-trolox and tacrine-tryptoline hybrids with different linker chain lengths were designed and synthesized. These hybrids demonstrated moderate to high AChE inhibitory activity (IC_50_: 17.37–2200 nM) and BuChE inhibitory activity (IC_50_: 3.16–128.82 nM) as well as free radical scavenging activities (IC_50_: 11.48–49.23 *μ*M). Docking experiments confirmed the binding of compounds to both the CAS and PAS of enzymes [58].

### 2.3. Synthetic Antioxidant Compounds in AD

Carbazole is naturally occurring and possesses an extensive range of biological activities used to develop anti-Alzheimer agents. The most potent multifunctional compound demonstrated effective and selective AChE inhibition, A*β* disaggregation, and metal chelation action. The radical scavenging assay of DPPH results showed 69.16 and 81.64% DPPH inhibition for the most active compounds [59]. Taj and coworkers also have been synthesized the benzamide derivatives which showed antioxidant and anti-Alzheimer activities. The most active compound in this series indicated the high inhibition of ACEs, as well as the highest scavenging percentage by DPPH assay [60].

Isoalloxazine derivatives are another class of compounds that have been evaluated for their neuroprotective potential by Machhi and coworkers. Based on AChE inhibitory activity, the most active compounds with good CNS penetration could improve cognitive ability in both scopolamine and A*β*1–42-induced Alzheimer's-like conditions in rodents. The antioxidant potential is confirmed by in vivo results. Additionally, these compounds could activate the Wnt/b-catenin pathway as evidenced by improved p-GSK-3, b-catenin, and neuroD1 levels in A*β*1–42-induced Alzheimer's rat brain [61]. The discovery of compounds with the ability to prevent the formation of free radicals in the brain is a beneficial strategy in the treatment of AD. Thus, synthesis and biological evaluation of 5-oxo-5H-furo[3,2-g]chromene-6-carbaldehydes derivatives against AChE and BChE has been reported by Mphahlele and coworkers. Various enzyme targets, such as *β*-secretase (BACE-1) and lipoxygenase-15 (LOX-15), as well as potential antioxidant are involved in AD. Evaluating the AChE and *β*-secretase inhibitory activity resulted in the most active compound in this series showing inhibitory effects, and it revealed modest antioxidant activity (IC_50_ = 7.4–23.9 *μ*M) relative to the ascorbic acid (IC_50_ = 4.8 *μ*M) [62]. Indole alkaloids are used as a vital pharmacological agent due to their biological effects. Yar and coworkers have assessed a series of selected synthetic indole derivatives to find new antioxidants via suppressing oxidative stress. DPPH scavenging assay for most of the compounds indicated significant to moderate antioxidant activity (IC_50_ value: 399.07–140.0 ± 50 *μ*M) [63].

As another example of the application of indole alkaloids in the design of cholinesterase inhibitors, Kanhed et al. made an effort to develop a series of indoloquinoxaline derivatives as multitarget-directed ligands. The indoloquinoxaline scaffold has been used as a promising entity that has stable binding affinities with both the AChE and BuChE enzymes [2, 3]. The synthesized compounds exhibited modest to good AChE inhibitory activity. The most active compound in this series inhibits the self-induced A*β*1–42 aggregation and possesses antioxidant activity [64].

Accumulation of the amyloid-beta peptide (A*β*) in neuritic plaques is connected to inflammatory responses due to the production of nitrosative stress that stimulates the expression of inducible nitric oxide synthase (iNOS). Increased nitric oxide leads to neuronal death and memory impairment. Therefore, treatment of AD using anti-inflammatory and antioxidant agents reduces the risk of developing AD. Guevara and coworkers assessed the effects of systemic administration of aminoguanidine (AG) (100 mg/kg/day for 4 days), as an iNOS inhibitor with anti-inflammatory and antioxidant properties, on spatial memory when inflammatory responses induced by an injection of A*β*25–35 [100 *μ*M] into the temporal cortex (TCx) of rats. AG treatment of the A*β*25–35-treated group improved spatial memory, which was linked with reductions in reactive gliosis, IL-1*β*, TNF-*α*, and nitrite levels, as well as in neurodegeneration in the TCx and hippocampus (Hp) [65].

**Figure 5:**
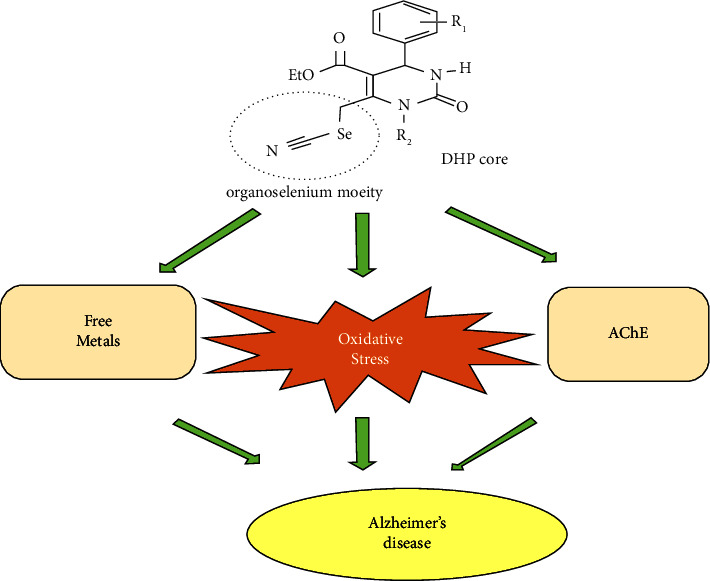
Structure of seleno-dihydropyrimidinones derivatives.

The effect of pure DDN (2,3-dichloro-5,8-dihydroxy-1,4-naphthoquinone) on the inhibition of AChE and A*β*42 aggregation was assessed, and the inhibition of A*β*42 aggregation (more than 90% at 25 *μ*M) and on AChE (IC_50_ = 14.5 *μ*M) was determined [66]. Inspired by the widespread use of triazine derivatives, Sinha et al. screened the novel triazine derivatives for their cholinesterase inhibition, and the most active compounds were selected for biological evaluation. The results confirmed the anticholinesterase and antioxidant activities of choosing compounds and indicated that they exerted their activities through decreasing in A*β*1–42 burden and cytochrome c as well as cleaved caspase-3 levels in the brain. Immunoblot and immunofluorescence data showed that these compounds could be able to improve the ratio of pGSK3/GSK3 and *β*-catenin which suggested positive involvement of the novel triazine derivatives in the Wnt/*β*-catenin pathway [67]. To develop potential multitargeted therapeutics, one may refer to the synthesis of seleno-dihydropyrimidinones compounds. As illustrated in Figure 5, for the design of novel AChE inhibitors, Braga and coworkers combined biologically active dihydropyrimidinones (DHPMs) and organoselenium compounds which have a vital role in modulating oxidative stress in the brain. All of the synthesized compounds demonstrated AChE inhibitory activity compared to the standard drugs while displaying good antioxidant activity via various mechanisms of action [68].

## 3. Analogues of Bioactive Compounds in AD

A potent antioxidant ferulic acid agent with antifibrillogenic and fibril-destabilizing effects against Aß was dimerized in order to increase its pharmacological efficiency against pathogenic A*β* oligomers. Results showed that one of the dimeric derivatives of ferulic acid, KMS4001, could decrease the A*β*1–40 and A*β*1–42 levels in the frontal cortex of APP/PS1 mutant transgenic mice [69]. Inspired by the important role of a bioactive compound Gx-50 for the treatment of AD, which is extracted from Sichuan pepper (*Zanthoxylum bungeanum*), various analogs like N-phenethyl cinnamide derivatives were designed and synthesized by Chai et al. Using a DPPH radical scavenging assay, antioxidant activities of synthesized compounds were evaluated. The most active compound could be able to inhibit the disaggregation of Cu^2+^-induced A*β*1–42 aggregation and indicate the potent antioxidative activity [70].

The irreversible fibril formation of various proteins, the main mechanism responsible for AD, has been introduced as an important target for developing anti-AD agents. Maity et al. reported a modified analog of curcumin, which possesses an antioxidant and antiamyloid activity aimed at improved stability. A pyrazole derivative of curcumin indicated notable potency in arresting the fibrillization of *β*-lactoglobulin (*β*-lg). Molecular docking revealed that such a result may be due to its interaction with aggregation-prone regions of the protein and prevention interactions between monomers, leading to suppression of the overall aggregation process [71].

Various studies have revealed that endogenous cannabinoid is implicated in the central nervous system and related diseases like AD. Valles and coworkers assessed the effect of WIN 55,212-2, a synthetic cannabinoid, on astrocytes function via the evaluation of different targets related to AD. This compound could be able to upregulate the expression of antioxidant enzyme Cu/Zn SOD and prevent inflammation induced by A*β*1-42 in cultured astrocytes through the increase of TNF-*α*, IL-1*β*, p-65, COX-2, and iNOS levels protein expression while reducing the expression of PPAR-*γ*. Hence, WIN 55,212-2 is an example of the beneficial effects of cannabinoids in the treatment of AD [72].

## 4. Other Synthetic Antioxidant Compounds in AD

Cholinesterase inhibitory, antioxidant, neuroprotective, and modulation of A*β* aggregation activities of flavone-8-acrylamide derivatives were evaluated by Amooru and coworkers. Modest antioxidant activity, strong neuroprotective capacities, accelerated A*β* aggregation, and selective inhibition of the AChE over BuChE was indicated by these novel multifunctional anti-Alzheimer agents [73]. Metal ions such as Cu, Fe, and Zn interacted with soluble and aggregated forms of A*β* peptide leading to the development of AD. Singh and coworkers synthesized the compound 2,6-bis[2-[(4-carboxyphenyl) methylene] hydrazide] as a novel Cu chelator and assessed its chelation ability in Cu-mediated neurotoxicity in the eye tissues of transgenic *Drosophila* expressing human amyloid *β*. Determination of antioxidant markers such as SOD and MDA showed a significant rescue [74].

Bukhari et al. synthesized a series of novel carbonyl compounds and evaluated their antioxidant activity and in vitro cytotoxicity. Most of the compounds were protective against A*β*-induced neuronal cell death in PC12 cells, and among them, the most active compound showed free radical scavenging activity (18.39 mM) and higher AChE inhibitory activity than donepezil [75]. Amyloid beta (A*β*) misfolding and aggregation are strongly implicated in AD. In pursuit of the development of aggregation modulators as effective therapeutic agents for AD, Ramesh et al. designed and synthesized a series of natural amino acids, L-dopa, and dopamine-appended derivatives of naphthalene diimide (NDI). Based on the results, L-dopa (NLD) and dopamine (NDP) conjugated NDIs could be able to modulate A*β*42-aggregation effectively and quench ROS [76].

Although a large number of compounds with antioxidant molecules have been synthesized, few have been successfully tested in clinical trials. This requires a better understanding of the mechanisms by which antioxidants work and where and when they are most effective. This understanding provides a rational approach that could lead to greater drug success in the future. However, the therapeutic use of synthetic molecules has largely been disappointing, largely due to inappropriate assumptions about how antioxidants work [77]. For example, scavenging the hydroxyl radical (•OH) is impractical, but preventing its formation by reducing the production of hydrogen peroxide (H_2_O_2_) can be effective in preventing damage. One of the misconceptions in this field is related to the removal of superoxide (O_2_^•−^) or H_2_O_2_ by small molecules [78]. This is because antioxidant enzymes react with those oxidants millions of times faster than small molecules, providing a dominant antioxidant defense [79]. The Mn porphyrin compounds have been synthesized, and some of them showed very high SOD activity [80]. Although whether the underlying mechanism is via SOD-like activity or another action remains elusive in some cases, the protective and therapeutic effects of many Mn porphyrins have been demonstrated in nonhuman animal models of diseases [81]. These preclinical results suggest the potential of Mn porphyrins in the clinical therapy of diseases [78]. Currently, a phase I clinical trial of MnTDE-2-ImP^5+^ in patients with amyotrophic lateral sclerosis showed no toxicity at therapeutic doses [82].

Although currently available treatments help manage the disease, they do not cure the disease [83, 84]. An important feature of Alzheimer's disease is oxidative stress. Despite evidence of the role of oxidative stress in the pathogenesis of Alzheimer's disease, none of the available treatment options have investigated oxidative stress [83]. Recently, important results of the effect of antioxidants in reducing the effects of oxidative stress on the central nervous system have been reported. Some antioxidants have proven positive effects on AD, but some still need attention and work [84]. Numerous studies and evidence show that oxidative damage is involved in the pathogenesis of AD through different pathways. Therefore, new therapeutic strategies for preventing oxidative damage may offer therapeutic efficacy against AD. Combining natural bioactivity with approved drugs could become an approach to prevent the onset of AD. An integrated system of antioxidants with several drugs may be more effective [84]. In addition, there is little information about the role of antioxidants in AD and the difference between the results of animal models and clinical trials. For this reason, there are doubts about the success of antioxidant therapy for AD. Investigating a more detailed approach to the connection between inflammation and AD and the integrated role of antioxidants in reducing inflammation should be considered [84]. Recently, the relationship between oxidative stress and the effect of natural antioxidants from natural sources and AD pathology has been discussed. This can lead to providing new therapeutic strategies.

It should be noted that the use of natural antioxidants has some limitations. Compared to synthetic antioxidants, natural antioxidants are mostly much more polar. Therefore, natural antioxidants (phenolic) are commonly not sufficiently soluble in the lipid phase [85]. This low solubility limits their efficiency in bulk lipids. Furthermore, in comparison to synthetic antioxidants with high purity (100%), natural antioxidants have a low degree of purity so a small part of the added composition is an antioxidant, and adding higher concentrations of natural antioxidants is necessary [85]. On the other hand, natural antioxidants are mainly less active and substrate specific. The antioxidant activity of natural antioxidants depends on synergists present both in the stabilized food and in the antioxidant preparation [86]. Other restrictions on the use of natural antioxidants are that these antioxidants possess certain toxicity so that too high concentrations of antioxidants are undesirable [87].

## 5. Conclusion

Alzheimer's disease (AD) is featured with cognitive dysfunction and memory lapse. Although the disease mechanism is yet to be properly characterized by various players such as deficit of acetylcholine (ACh), oxidative stress inflammation, *β*-amyloid (A*β*) deposits, and dyshomeostasis of biometals are involved in the progress of AD. Multiple pieces of evidence indicated that intracellular ROS with an important role in the pathogenesis of AD increased in brains' AD. ROS could be able to damage a wide range of critical biomolecules related to neurodegenerative disease and lead to neuronal cell death. The antioxidant compounds have been considered an effective therapeutic strategy. In this review, compounds with antioxidant activity that have played a role in AD are classified into three classes. Most of the investigated antioxidant reagents were obtained from natural products as one of the main classes of antioxidants. The majority of natural product-based antioxidants were phenolic compounds that could act as hydrogen donors or chelate metal ions and inhibit the oxidation of low-density lipoproteins (LDL) leading to a decrease in risks of neurodegenerative diseases. Hybrid compounds, like other classes of compounds, were designed in order to have antioxidant and anti-AD activities in a single entity. The last classes of antioxidant compounds related to AD were synthetic reagents with different structures. Considering these three classes of compounds, information is provided for further direction of anti-AD compound design. Most of the investigated compounds in the current paper target a variety of pathways and proteins involved in AD. This multitargeted feature provides useful benefits to tackle the intertwined pathogenesis of AD. Collectively, using antioxidant compounds in the treatment of AD due to their significant outcomes serves as a promising strategy.

## Data Availability

All data generated or analyzed during this study are included in this published article.
